# StressSpeak: A Speech-Driven Framework for Real-Time Personalized Stress Detection and Adaptive Psychological Support

**DOI:** 10.3390/diagnostics15222871

**Published:** 2025-11-12

**Authors:** Laraib Umer, Javaid Iqbal, Yasar Ayaz, Hassan Imam, Adil Ahmad, Umer Asgher

**Affiliations:** 1 School of Mechanical and Manufacturing Engineering, National University of Sciences and Technology, Islamabad 44000, Pakistan; 2 National Center of Artificial Intelligence, National University of Sciences and Technology, Islamabad 44000, Pakistan; 3 Department of Optometry and Orthoptics, Faculty of Rehabilitation and Allied Health Sciences, Riphah International University, Islamabad 44000, Pakistan; 4 School of Interdisciplinary Engineering and Sciences (SINES), National University of Sciences and Technology (NUST), Islamabad 44000, Pakistan; 5 Laboratory of Human Factors and Automation in Aviation, Department of Air Transport, Faculty of Transportation Sciences, Czech Technical University in Prague (CTU), 128 00 Prague, Czech Republic

**Keywords:** real-time stress detection, large language models (LLMs), personalized digital interventions, speech-based emotion recognition, stress classification, clinical decision support

## Abstract

**Background:** Stress is a critical determinant of mental health, yet conventional monitoring approaches often rely on subjective self-reports or physiological signals that lack real-time responsiveness. Recent advances in large language models (LLMs) offer opportunities for speech-driven, adaptive stress detection, but existing systems are limited to retrospective text analysis, monolingual settings, or detection-only outputs. **Methods:** We developed a real-time, speech-driven stress detection framework that integrates audio recording, speech-to-text conversion, and linguistic analysis using transformer-based LLMs. The system provides multimodal outputs, delivering recommendations in both text and synthesized speech. Nine LLM variants were evaluated on five benchmark datasets under zero-shot and few-shot learning conditions. Performance was assessed using accuracy, precision, recall, F1-score, and misclassification trends (false-negatives and false-positives). Real-time feasibility was analyzed through latency modeling, and user-centered validation was conducted across cross-domains. **Results:** Few-shot fine-tuning improved model performance across all datasets, with Large Language Model Meta AI (LLaMA) and Robustly Optimized BERT Pretraining Approach (RoBERTa) achieving the highest F1-scores and reduced false-negatives, particularly for suicide risk detection. Latency analysis revealed a trade-off between responsiveness and accuracy, with delays ranging from ~2 s for smaller models to ~7.6 s for LLaMA-7B on 30 s audio inputs. Multilingual input support and multimodal output enhanced inclusivity. User feedback confirmed strong usability, accessibility, and adoption potential in real-world settings. **Conclusions:** This study demonstrates that real-time, LLM-powered stress detection is both technically robust and practically feasible. By combining speech-based input, multimodal feedback, and user-centered validation, the framework advances beyond traditional detection only models toward scalable, inclusive, and deployment-ready digital mental health solutions.

## 1. Introduction

### 1.1. Background

Stress is a pervasive factor contributing to a wide range of physical and mental health disorders, including headaches, cardiovascular diseases, anxiety, and depression [1,2]. The increasing societal burden of stress highlights the urgent need for effective and timely detection and management strategies [3]. However, conventional stress monitoring techniques largely rely on subjective self-reports or physiological measurements, both of which present notable limitations. Self-reported data can be inconsistent and unreliable, as individuals may be unaware of their own emotional states or unable to articulate them accurately [4,5]. Physiological signals, such as heart rate variability and skin conductance, though objective, often fail to capture the evolving and context-sensitive nature of stress in real-world settings [6,7].

Given these challenges, there is a growing interest in developing AI-driven approaches to enhance stress detection capabilities.

Artificial intelligence (AI), particularly natural language processing (NLP), offers new avenues for stress detection by analyzing linguistic markers in speech and writing. Large Language Models (LLMs), a significant advancement in artificial intelligence, have demonstrated remarkable proficiency in understanding and generating human language [8]. Trained on vast corpora of text data, LLMs can detect nuanced patterns associated with emotional states, including stress [9]. These models have recently shown remarkable ability to detect subtle emotional patterns in language [8,9]. Unlike static text analyses, speech-driven approaches have the potential to capture stress in real time.

### 1.2. Research Gap

Traditional methods that rely on self-reports or physiological proxies frequently lack real-time responsiveness, limiting their utility in dynamic environments [10,11]. The emergence of natural language processing (NLP) methods offers new possibilities by analyzing the linguistic expression of stress in speech or writing. Studies have shown that the language individuals use, such as the prevalence of negative words, disrupted sentence structure, or accelerated speech rate, can serve as strong indicators of emotional strain [12,13]. Nevertheless, most prior work has focused on analyzing static text sources, such as social media posts, which capture stress retrospectively rather than in the moment or real-time assessment.

### 1.3. Objectives of the Study

To address this gap, this study proposes a real-time stress detection system that captures spoken audio, transcribes it into text, and analyzes the linguistic markers of stress using LLMs [14,15]. By examining features such as word choice, tone, and sentence organization, the system classifies stress levels into mild, moderate, and severe categories [16,17], enabling a more detailed and immediate understanding of an individual’s emotional state [18,19].

In addition to detection, the system provides tailored, actionable recommendations based on the identified stress level [20]. For instance, users classified with moderate stress may be encouraged to engage in breathing exercises, take mindful breaks, or listen to calming music, whereas severe cases may be guided toward more structured interventions. These personalized suggestions are designed to be practical, easily implementable, and context-aware, aiming to reduce stress in real time [21].

The use of LLMs in this context introduces a new dimension to stress management by combining linguistic analysis with psychological assessment principles [22,23]. This integration allows for a more accurate, accessible, and effective approach to understanding and managing emotional well-being.

Through this research, we aim to significantly contribute to the growing body of literature on AI applications in mental health, providing a foundation for future advancements in real time, responsive emotional support technologies [24,25].

The objectives of this study were:

- To design and implement a real-time stress detection system based on large language models (LLMs), integrating audio recording, speech-to-text conversion, and multimodal input–output capabilities (text and synthesized speech).
- To evaluate and compare transformer-based models across multiple benchmark stress-related datasets, analyzing accuracy, precision, recall, F1-score, and error rates in both zero-shot and few-shot learning settings.
- To examine real-time feasibility by quantifying latency–accuracy trade-offs across different model sizes and audio input lengths.
- To validate system usability and acceptance in real-world contexts through real-time user feedback from cross-domains.

By combining linguistic analysis with psychological principles, the framework seeks to move beyond detection-only systems and deliver context-aware, personalized digital interventions [22,23].

## 2. Related Work

### 2.1. Language-Based Stress Detection

Online platforms, particularly social media, have increasingly been recognized as valuable resources for understanding individuals’ psychological states, health behaviors, and overall well-being [26,27]. Over the past decade, extensive research has explored the use of content analysis and social interaction patterns to predict risks associated with mental health conditions such as anxiety, depression, and suicidal ideation [28,29]. The archival nature of social media provides opportunities to track psychological risk factors over time, while minimizing self-reporting biases [30,31].

Early investigations into mental health monitoring primarily employed basic linguistic analysis techniques to examine correlations between online language and psychological conditions [32,33].

### 2.2. Machine Learning and Deep Learning Approaches

As research progressed, more sophisticated machine learning and deep learning models were introduced to enhance predictive capabilities [34,35,36]. For example, Support Vector Machines (SVMs) were utilized to predict depressive symptoms, while architectures such as Long Short-Term Memory (LSTM)- Convolutional Neural Networks (CNNs) networks were deployed for detecting suicidal ideation on platforms like Reddit [37,38].

### 2.3. Large Language Models (LLMs) in Mental Health Prediction

The recent advent of pre-trained language models, such as Bidirectional Encoder Representations from Transformers (BERTs), has revolutionized mental health prediction tasks by providing models trained on massive corpora and subsequently fine-tuned for specific applications [39,40]. Additionally, multitask learning approaches have emerged, allowing models to simultaneously predict multiple mental health conditions [41,42], although such systems often remain constrained to fixed task sets [43,44]. Building on this foundation, the current research aims to explore next-generation LLMs fine-tuned with specific instructions, assessing their potential in mental health prediction across diverse and dynamic data sources.

Following the success of Transformer-based architectures like BERT and Generative Pre-Trained Transformers (GPTs), research and industry have increasingly focused on developing larger, more capable models such as GPT-3 and Text-to-Text Transfer Transformer (T5) [45]. Techniques such as instruction fine-tuning have further expanded model versatility by exposing LLMs to diverse datasets and prompt types, enabling generalized task adaptation [46,47]. Models such as GPT-4, Pathways Language Model (PaLM), Fine-Tuned Language Net—T5 (FLAN-T5), LLaMA, and Alpaca, each containing tens to hundreds of billions of parameters, have demonstrated state-of-the-art performance in a variety of tasks, including question answering, logical reasoning, and machine translation [48,49].

Recent efforts have also explored the application of LLMs in healthcare settings [50]. For instance, models like PaLM-2 and LLaMA have been fine-tuned on medical datasets, achieving promising results in clinical question answering and decision support tasks [49,51]. However, the use of LLMs in the mental health domain remains relatively underexplored. Early studies evaluated LLMs such as ChatGPT (GPT-3.5) for tasks involving sentiment analysis, emotional reasoning, and preliminary mental health assessments [52]. Despite showing considerable promise, existing models exhibit notable performance gaps, typically 5–10% lower in accuracy and F1-score, when applied to complex emotional prediction tasks [53].

More recently, specialized models such as Mental-LLaMA, fine-tuned specifically on mental health datasets, have been introduced. However, these efforts have primarily focused on limited models (e.g., LLaMA or GPT-3.5) and mostly explored zero-shot learning paradigms without systematically investigating alternative techniques for performance enhancement. In this context, the present research comprehensively explores the potential of multiple LLMs for real-time mental health prediction, with a focus on refining their capabilities through task-specific fine-tuning and instruction-based optimization.

## 3. Methodology

### 3.1. System Overview

**Figure 1:**
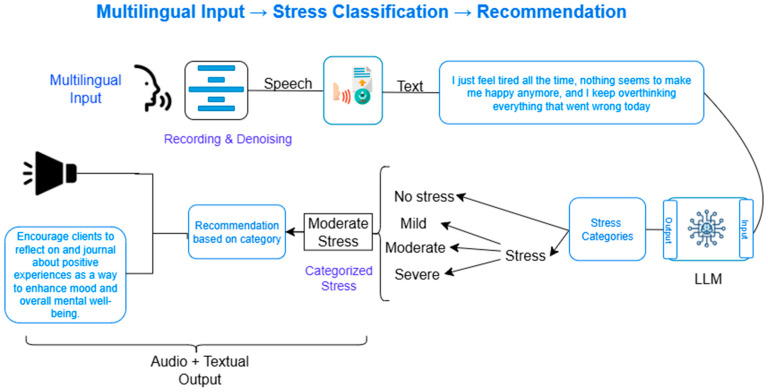
A schematic overview of the end-to-end system workflow. (Multilingual input 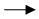 Speech 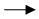 Text conversion 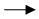 LLM 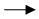 Stress detection and its classification 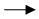 Recommendation based on categorized stress level 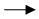 Audio + Textual Output).

This research proposes a comprehensive system for real-time stress detection and personalized management by integrating audio signal processing, natural language analysis, and advanced large language modeling (LLM) techniques. The methodological pipeline consists of five primary stages as shown in Figure 1: audio acquisition, speech-to-text conversion, text preprocessing, stress analysis using LLMs, and personalized recommendation generation.

### 3.2. Audio Acquisition and Speech-to-Text Conversion

The system initiates by capturing real-time audio inputs through microphone-enabled devices such as smartphones, tablets, or computers. Users are prompted to describe their feelings, experiences, or current emotional states verbally. To ensure high-fidelity transcription, a state-of-the-art speech-to-text (STT) engine is employed, capable of handling diverse accents, variable speech rates, and background noise conditions.

The transcribed text forms the basis for subsequent linguistic and emotional analysis.

### 3.3. Text Preprocessing

The textual data undergoes a multi-stage preprocessing pipeline to enhance quality and readiness for analysis:

- Noise Removal: Elimination of non-informative components such as filler words, long pauses, or repeated phrases.
- Normalization: Standardization procedures including lowercasing, punctuation correction, and removal of extraneous characters.
- Error Correction: Correction of potential transcription inaccuracies using linguistic and grammatical tools.
- Tokenization: Division of text into structured units (tokens) suitable for input into large language models.

This preprocessing ensures that the input accurately captures the user’s linguistic expressions while minimizing artifacts.

### 3.4. Stress Analysis Using Large Language Models

The preprocessed text is analyzed by a fine-tuned LLM trained for sentiment detection and stress classification. The model examines multiple linguistic dimensions, including:

- Lexical choice (e.g., prevalence of negative language)
- Syntactic structure (e.g., sentence complexity, disruptions)
- Emotional tone and semantic coherence

Based on these features, the system classifies the user’s emotional state into three stress levels:

- Mild Stress
- Moderate Stress
- Severe Stress

The classification enables nuanced, real-time monitoring of emotional well-being.

### 3.5. Personalized Recommendation Generation

Following stress classification, the system delivers actionable interventions tailored to the user’s current emotional state. Recommendations may include:

- **Mild Stress:** Reflective journaling, gratitude exercises, or short mindful activities.
- **Moderate Stress:** Structured breathing exercises, mindfulness practices, or guided relaxation.
- **Severe Stress:** Encouragement to seek professional mental health support, crisis helpline resources, or urgent self-care strategies.

The personalized feedback mechanism aims to provide immediate emotional support and promote proactive stress management.

### 3.6. Web-Based User Interface

**Figure 2:**
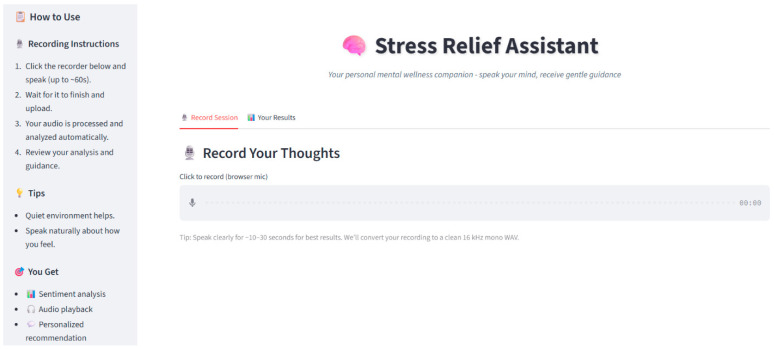
Screenshot of User-Interface (UI) for user engagement.

**Figure 3:**
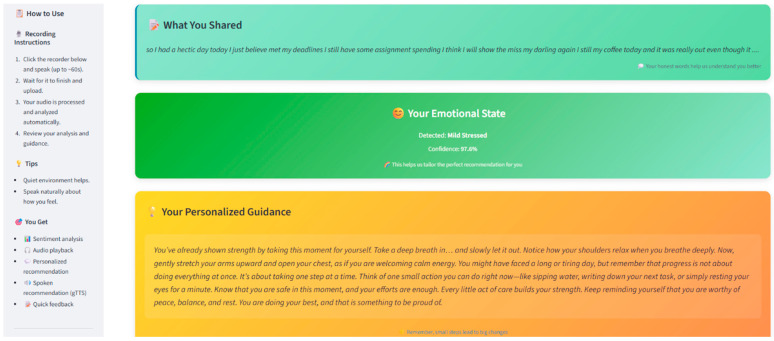
Screenshot of working User-Interface (UI).

To ensure accessibility and ease of interaction, a web-based user interface (UI) has been developed, screenshot of UI is depicted in Figure 2 and Figure 3. Figure 2 shows the user interface while Figure 3 shows the working of user interface.

Key features of the interface include:

- Real-time audio recording and transcription display
- Visualization of emotional and stress analysis results
- Delivery of personalized, dynamic intervention suggestions

The intuitive design ensures that users across diverse backgrounds can seamlessly access real-time stress detection and management services through internet-enabled devices.

### 3.7. Validation of the Proposed Model Using Benchmark Datasets

The validation of the proposed real-time stress detection system was conducted using five benchmark datasets, each targeting different dimensions of stress and mental health assessment. The evaluation process was structured around two learning paradigms: zero-shot learning and few-shot learning. The primary objective was to assess the model’s generalization capabilities and its adaptability to task-specific nuances using limited labeled data. In addition to benchmark dataset evaluation, a real-time pilot usability test was conducted with 25 voluntary participants, including students, educators, and healthcare professionals. Participants were recruited via convenience sampling (probability method, i.e., simple random sampling). Since this was an exploratory usability validation, no formal sample size calculation was performed. The focus was to assess practical feasibility and user experience rather than hypothesis testing.

### 3.8. Benchmark Datasets

The datasets selected for validation provided comprehensive coverage across social, clinical, and high-risk emotional contexts:

- Stress Annotated Dataset (SAD): Focused on identifying social anxiety from online forum posts [54].
- Dreaddit: A Reddit-based dataset annotated for varying stress levels [55].
- DepSeverity: A dataset measuring the severity of depressive symptoms [56].
- Suicide Depression Classification with Noisy Labels (SDCNL): Counseling transcript data annotated for stress and emotional distress [57].
- Columbia-Suicide Severity Rating Scale (CSSRS)-Suicide: A dataset derived from the Columbia-Suicide Severity Rating Scale to classify suicidal risk levels [58].

The diversity of these datasets ensured a robust and multi-dimensional evaluation of the model’s capabilities.

### 3.9. Learning Paradigms

Two learning paradigms were employed:

- Zero-Shot Learning: The model was evaluated without any task-specific fine-tuning, relying purely on pre-trained knowledge representations. This assessed the system’s inherent ability to generalize to unseen domains.
- Few-Shot Learning: The model underwent fine-tuning using a limited number of labeled examples from each dataset. This approach evaluated the model’s adaptability and its capacity to learn task-specific features from minimal data exposure.

## 4. Experimental Results

The proposed system’s performance was benchmarked against several established models, including Gemma, BERT, RoBERTa, DistilBERT, XLNet, T5, DeBERTa, and Electra [59,60].

Key observations include:

- LLaMA consistently demonstrated competitive or superior performance across most datasets.
- While RoBERTa slightly outperformed LLaMA on SAD after fine-tuning, LLaMA showed better robustness on diverse and more challenging datasets like SDCNL and CSSRS-Suicide.

**Figure 4:**
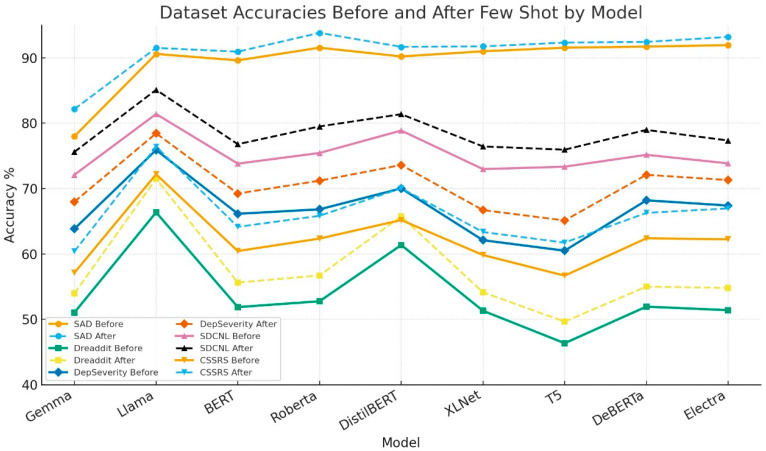
Comparison of Performance Plot Before and After Fine-Tuning Across Datasets.

These findings emphasize the robustness of the proposed system, especially under real-world noisy data conditions. The performance results are summarized in Figure 4, detailing accuracy values across all datasets before and after fine-tuning. Figure 4 graphically illustrates the model’s performance plot improvements with zero-shot learning and few-shot learning.

### 4.1. Comprehensive Metric Evaluation

To comprehensively assess the robustness of the proposed system, we evaluated nine transformer-based models (Gemma, LLaMA, BERT, RoBERTa, DistilBERT, XLNet, T5, DeBERTa, and Electra) across five benchmark datasets: SAD, SDCNL, CSSRS-Suicide, DepSeverity, and Dreaddit. Each model was tested under both zero-shot and few-shot paradigms. Performance metrics included Accuracy, Precision, Recall, F1-score, along with False Negative (FN) and False Positive (FP) rates, thereby providing a balanced evaluation of predictive capability and error tendencies. This approach provided a multi-dimensional view of performance, capturing not only predictive capability but also the reliability of classification under different conditions. Table 1 shows the model performance on SAD dataset including performance metrics like accuracy, precision, recall, F1-score, along with false negative (FN) and false positive (FP) rates.

On the SAD, LLaMA and RoBERTa achieved the strongest results, with RoBERTa reaching 93.8% accuracy and balanced precision-recall in the few-shot setting. DistilBERT and DeBERTa also performed competitively, whereas Gemma lagged behind with significantly lower scores. Few-shot fine-tuning notably reduced FN and FP rates across all models, improving robustness. Table 2 shows the model performance on SDCNL dataset including performance metrics like accuracy, precision, recall, F1-score, along with false negative (FN) and false positive (FP) rates.

In the SDCNL dataset (counseling transcripts), LLaMA again showed superior performance, improving from 81.4% to 85.1% accuracy after few-shot fine-tuning. DistilBERT achieved competitive recall (81.4%), but with slightly higher FP rates. Overall, models demonstrated consistent improvements with few-shot training, indicating adaptability to structured conversational data. Table 3 shows the model performance on CSSRS-Scuicide dataset including performance metrics like accuracy, precision, recall, F1-score, along with false negative (FN) and false positive (FP) rates.

The CSSRS-Suicide dataset posed the greatest challenge due to its high-risk nature. LLaMA delivered the highest performance with an F1-score of 84.54% after fine-tuning, followed closely by RoBERTa and DeBERTa. Smaller models such as Gemma and T5 showed weaker results, with high FN rates that could risk under-detection. This highlights the importance of larger models for sensitive clinical tasks. Table 4 shows the model performance on DepSeverity dataset including performance metrics like accuracy, precision, recall, F1-score, along with false negative (FN) and false positive (FP) rates.

In DepSeverity, LLaMA outperformed others, achieving 78.4% accuracy with improved F1 and reduced FN rates in the few-shot setup. BERT and RoBERTa demonstrated moderate gains but remained below LLaMA. DistilBERT offered reasonable precision and recall balance, while Gemma consistently underperformed. Table 5 shows the model performance on Dreaddit dataset including performance metrics like accuracy, precision, recall, F1-score, along with false negative (FN) and false positive (FP) rates.

The Dreaddit dataset (social media posts) was the most noisy and unstructured. Baseline performance was generally low, with most models below 60% accuracy in the zero-shot setting. After fine-tuning, LLaMA improved to 71.5% accuracy, outperforming all others. DistilBERT showed solid improvements as well, while Gemma and T5 had the weakest results. This reinforces that fine-tuning is critical for handling noisy, real-world text.

Summary of findings across all datasets:

- Few-shot fine-tuning consistently boosted performance, lowering FN/FP rates.
- LLaMA and RoBERTa emerged as the most reliable models, especially for high-risk and structured datasets.
- Smaller models (Gemma, T5) underperformed, making them less suitable for stress-sensitive tasks.
- The trade-off between model size, accuracy, and latency (as noted in the latency analysis) is crucial when considering real-time deployment.

### 4.2. Real-Time Performance Evaluation

To evaluate practical feasibility, we tested the system in real-time conditions with spoken input from individual participants.

#### 4.2.1. Latency Analysis

Latency is a critical factor in evaluating the real-time applicability of stress detection systems. Our analysis indicates that latency grows with both audio length and model size. Processing longer audio requires handling more frames, while larger models perform more computation per frame, leading to increased delays.

Using a simple estimate for a 30 s audio clip, latency can be approximate as:

Latency (seconds) = 2.0 + (0.8 × Model Size in Billions of Parameters)

**Figure 5:**
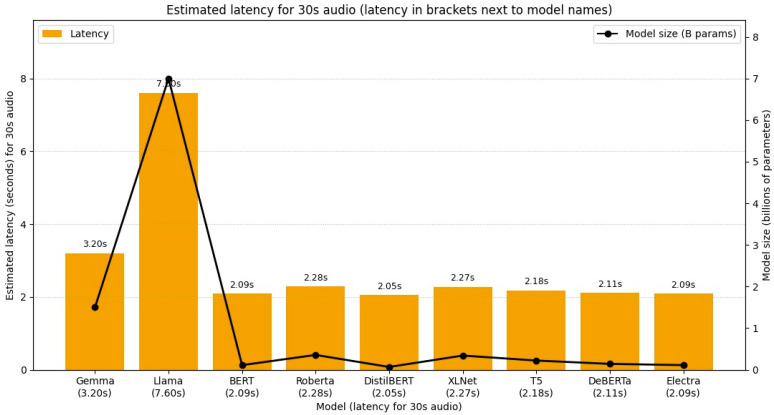
Illustrates latency distribution and overall real-time performance trends.

Based on this formula, latency values range from approximately 2.05 s for a distilled model to 7.60 s for LLaMA 7 billion. This trade-off underscores the balance between achieving high classification accuracy and maintaining responsiveness in real-time applications. Figure 5 illustrates latency distribution and overall real-time performance trends.

#### 4.2.2. Multilingual Support

The speech recognition and LLM modules demonstrated the ability to process inputs in multiple languages. This indicates that the system is robust across diverse linguistic contexts, an essential feature for real-world deployment where users may not always communicate in English. Future work will expand this capability by incorporating training data from additional languages and dialects to further improve accuracy and inclusivity.

#### 4.2.3. Input and Output Modes

The system successfully received input in speech and generated responses in both text and synthesized speech formats. The input and output of the system did not store anywhere, therefore, removes privacy concerns as well. This dual-mode functionality enhances accessibility by ensuring that users with different preferences and needs, such as those with visual impairments or literacy barriers, can still benefit from real-time stress detection and intervention. Delivering feedback in multiple modalities also improves user engagement and adaptability in various use cases.

### 4.3. Case Studies from Benchmark Datasets

To further validate system performance, representative instances from each dataset were analyzed. The model not only classified the stress levels correctly but also generated contextually appropriate personalized recommendations.

Examples include:

- In SAD, expressions of social anxiety were classified as moderate stress, prompting journaling and exposure-therapy suggestions.
- In CSSRS-Suicide, signs of suicidal ideation were flagged, and immediate intervention recommendations were generated.

These examples, summarized in Table 6, illustrate the practical applicability of the system in providing real-time emotional support.

### 4.4. User Feedback from Different Domains

To complement quantitative evaluation, we gathered user feedback from individuals across multiple domains who interacted with the real-time stress detection system. Feedback analysis highlighted the following trends:

- Healthcare professionals appreciated the potential for non-invasive monitoring but emphasized the importance of clear disclaimers regarding clinical use.
- Students and educators valued multilingual processing and immediate coping suggestions, reporting increased engagement compared to static self-report tools.
- Corporate employees particularly favored the speech-based interface for quick stress check-ins during work hours, citing improved accessibility over text-only methods.

**Figure 6:**
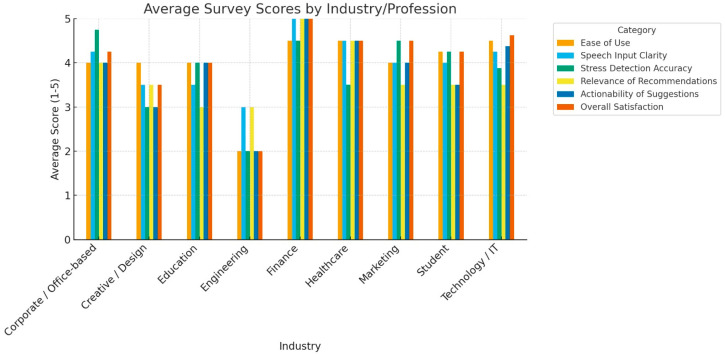
Average user feedback summary across multiple professional domains (healthcare, education, and corporate). The figure illustrates overall satisfaction with system usability, accessibility, multilingual support, and effective intervention, highlighting consistent positive reception across diverse user groups.

Graphical summary of this feedback is provided in Figure 6. Overall, users reported that the system was intuitive, accessible, and responsive.

## 5. Discussion

This Discussion is structured around the four objectives of the study: (i) to design and implement a real-time stress detection system integrating audio acquisition and multimodal outputs; (ii) to evaluate transformer-based LLMs across multiple stress-related datasets; (iii) to examine latency–accuracy trade-offs in real-time conditions; and (iv) to validate system usability through cross-domain user feedback.

This study introduced a real-time, speech-driven stress detection and management system powered by large language models (LLMs), addressing critical gaps left by previous approaches that were often retrospective, text-only, or monolingual [4,5,6,7,11,52]. By leveraging few-shot fine-tuning, the system achieved robust performance across benchmark datasets, notably improving recall and F1 in high-risk contexts such as suicidal ideation detection [61]. Beyond accuracy, the system demonstrated practical feasibility by analyzing latency-accuracy trade-offs [62], supporting multilingual inputs, and providing dual output modes. User-centered validation underscored strong usability, highlighting its readiness for real-world applications.

This study advances the field of computational mental health by presenting and validating a real-time stress detection and management system that integrates speech recognition, text preprocessing, and fine-tuned large language models (LLMs). The results across five benchmark datasets and real-time evaluations provide compelling evidence for both the technical robustness and the practical feasibility of the proposed approach. The following subsections analyze and interpret the results in relation to these objectives.

### 5.1. LLM Robustness Across Diverse Contexts

The comparative evaluation across nine transformer-based architectures demonstrates that larger LLMs (LLaMA, RoBERTa, DeBERTa) consistently outperform smaller counterparts such as Gemma and T5, particularly on high-risk datasets like CSSRS-Suicide. This is not surprising, as larger models leverage broader contextual representations, enabling them to capture subtle linguistic markers of stress and suicidality. Importantly, the few-shot learning paradigm proved indispensable, markedly reducing false negatives and false positives across all datasets. From a clinical perspective, reducing false negatives is crucial because under-detection of severe stress or suicidal ideation may carry significant risks. The superior performance of LLaMA (F1 = 84.54% on CSSRS-Suicide after few-shot tuning) therefore underscores the potential of such models in sensitive, safety-critical applications.

### 5.2. Trade-Offs Between Model Size, Accuracy, and Latency

The latency analysis highlights the practical tension between computational cost and predictive performance. While distilled or smaller models deliver near-instantaneous results (~2 s), their weaker accuracy renders them less suitable for clinical or high-stakes use. Conversely, larger models such as LLaMA-7B, despite yielding the highest accuracies, introduce latencies up to ~7.6 s for a 30 s clip. In real-world contexts, such delays are acceptable for self-monitoring applications but may hinder high-frequency workplace monitoring or clinical triage. This trade-off suggests that hybrid deployment strategies, using lightweight models for low-stakes screening and larger models for high-risk or flagged cases, could maximize both responsiveness and reliability.

### 5.3. Multilingual and Multimodal Strengths

The system’s ability to handle multilingual speech inputs and to provide dual-mode outputs (text and synthesized speech) is a critical innovation. These capabilities extend accessibility to diverse populations, including non-English speakers, visually impaired individuals, and those with literacy challenges. Unlike prior stress detection systems limited to text-only inputs or single-language corpora, [33,38], this approach demonstrates scalable inclusivity, making it adaptable for deployment in multilingual societies and global mental health initiatives.

### 5.4. User-Centered Evaluation

The incorporation of user feedback across professional domains adds a practical validation layer often missing in computational mental health studies. Healthcare professionals highlighted the promise of non-invasive monitoring but also raised the need for clear clinical disclaimers, a caution against premature medicalization. Educators and students valued the immediacy of multilingual feedback and coping suggestions, aligning with the push for mental health support in educational institutions. Corporate users praised the ease of speech-based interaction, reinforcing the system’s potential in workplace well-being programs. Importantly, the consistently positive reception across groups indicates strong usability and social acceptability, which are essential for real-world adoption.

### 5.5. Comparative Advantages over Previous Studies

1. Integration of Real-Time Speech + LLMs vs. Retrospective Text-Based Methods: Many earlier stress-detection systems focus on textual or physiological signals retrospectively but do not include real-time speech input [4,5,6,7,11]. Moreover, while several studies have developed chatbot systems for mental health [52], our model extends beyond static conversational agents by integrating stress-level classification and adaptive response generation. This aligns with calls for more intelligent, context-aware digital interventions in psychological care [48,50]. However, our system integrates live audio acquisition, speech-to-text conversion, and immediate stress classification, making detection timely and context-aware.
2. Multi-Metric Evaluation (Accuracy, FN/FP, Precision, Recall, F1) vs. Accuracy-Only or Binary Metrics: Several studies report only accuracy or binary classification (stress vs. non-stress) and often without detailed error analysis for false negatives/positives [63]. Our work goes further by examining FN rate, FP rate, precision, recall, and F1 in both zero-shot and few-shot settings across multiple datasets, providing a more nuanced view of model reliability, especially important in sensitive contexts such as suicidal ideation detection.
3. Few-Shot Fine-Tuning Improves Generalization vs. Models Requiring Large Labeled Data: Traditional machine learning (ML) and deep learning (DL)-based methods (e.g., using Support Vector Machine (SVM), Convolutional Neural Network (CNN), Recurrent Neural Network (RNN)) often require substantial annotated data for each domain or dataset [64]. Our study shows that with few-shot fine-tuning, even with limited new examples, performance improves significantly across datasets. This suggests better adaptability to new domains, and less dependence on large annotation efforts.
4. Multilingual Support and Dual Output Modes vs. Monolingual, Text-Only Systems: Many previous works assume English input or text only [65]. Our model supports multilingual speech and outputs in both text and synthesized speech. This improves accessibility (for non-English users, those who prefer voice feedback), an important advance over prior systems that were more constrained.
5. Latency Analysis & Real-Time Feasibility vs. High Accuracy Alone: Some prior work achieves high accuracy but do not report or consider latency or real-time usability. For example, many machine learning (ML)/Deep Learning (DL) methods in the literature focus purely on feature extraction + classification (speech signal, physiological data) without considering audio length/model size trade-offs [61]. We provide a latency model (Latency = 2.0 + 0.8 × model size in billions), highlighting how larger models incur longer delays. This allows for a practical assessment of trade-offs required for deployment in real-world scenarios.
6. Domain Diversity and Risk-Sensitive Datasets vs. Simpler Data: Several studies use relatively clean datasets or those not involving high risk (e.g., general stress, depression). Our validation includes CSSRS-Suicide dataset, which addresses suicidal ideation, a high-risk domain where false negatives have severe consequences. The performance on CSSRS-Suicide (with few-shot fine-tuning) demonstrating high recall and F1 is an important strength.

Having compared our system with prior work, we next discuss its broader positioning against the state of the art and the implications for clinical and practical deployment

### 5.6. Positioning Against State of the Art

Relative to existing literature, the present study makes three strong contributions:

1. It establishes that few-shot fine-tuned LLMs outperform both traditional ML and smaller transformer baselines in detecting stress across varied linguistic contexts.
2. It introduces multilingual, multimodal, real-time capabilities, bridging a critical gap in accessibility and inclusivity.
3. It validates not only algorithmic performance but also human-centered usability across professional domains, strengthening the case for real-world deployment.
4. The input and output of the system do not store and therefore removes privacy concerns as well.

### 5.7. Limitations

While the findings are promising, several methodological and practical limitations must be acknowledged to assess both internal and external validity. Taken together, these findings argue for a paradigm shift in stress monitoring from retrospective, text-only analysis toward real-time, speech-driven, LLM-powered systems. The improvements demonstrated with few-shot learning suggest that future systems can be rapidly adapted to domain-specific environments. However, several limitations warrant consideration. First, the real-time evaluation involved a modest sample size (*n* = 25) using probability (simple random) sampling, which restricts external validity. Second, latency constraints highlight the need for edge-optimized LLMs that preserve accuracy without compromising speed. Third, although performance improved with few-shot fine-tuning, zero-shot results remain weaker, which may limit generalizability without domain-specific adaptation. Fourth, while multilingual support was tested, only a subset of languages and dialects were covered, requiring further cultural and linguistic validation. Fifth, multilingual support was tested, but not all languages/dialects were represented, further cultural/linguistic validation is needed. Sixth, the study was conducted under controlled usability settings and did not include longitudinal financial or access-related feasibility studies.

### 5.8. Future Directions

Building on these findings and limitations, the following directions are proposed for future research and system development.

- Extend evaluation to more languages, dialects, and speech styles (slang, colloquial speech).
- Explore on-device or edge computing strategies to reduce latency while preserving accuracy.
- Incorporate additional modalities (e.g., facial expression, physiological signals) to strengthen detection and reduce false negatives in high-risk categories.
- Conduct larger-scale longitudinal validation across diverse populations to evaluate the sustained effectiveness, reliability, and clinical impact of the system over extended periods of real-world use.

## 6. Conclusions

This study introduced a real-time, speech-driven stress detection and management system powered by large language models (LLMs), addressing critical gaps left by previous approaches that were often retrospective, text-only, monolingual, or focused solely on accuracy. By leveraging few-shot fine-tuning, the system achieved robust performance across five benchmark datasets, with notable improvements in reducing false negatives for high-risk contexts such as suicidal ideation detection.

Beyond technical robustness, the system demonstrated practical feasibility by analyzing latency-accuracy trade-offs, supporting multilingual speech inputs, and providing dual output modes (text and synthesized speech). Importantly, it incorporated user feedback from healthcare, education, and corporate domains, underscoring strong usability and acceptance, an aspect rarely emphasized in prior work.

The findings highlight that LLMs, when carefully adapted, can provide accurate, inclusive, and responsive solutions for mental health monitoring. This positions our work as a deployment-ready framework rather than a purely experimental system.

Nevertheless, challenges remain. Performance under zero-shot settings on noisy data requires further improvement and latency optimization is needed for large-scale deployment is essential. Future research should explore edge-optimized deployment, integration of multimodal signals (e.g., physiological or visual), and longitudinal clinical studies to assess sustained real-world impact.

In conclusion, this study provides evidence that real-time, LLM-powered stress detection systems represent a transformative step forward in mental health technology, offering not only technical advancement but also practical pathways for safe, inclusive, and scalable adoption across diverse domains.

## Figures and Tables

**Table 1:** Performance of Models on SAD (Accuracy, FN rate, FP rate, Precision, Recall, and F1-score in Zero-Shot and Few-Shot learning).

Phase	Metric	Gemma	Llama	BERT	Roberta	DistilBERT	XLNet	T5	DeBERTa	Electra
Zero-Shot	Accuracy	78	90.6	89.6	91.5	90.2	91	91.5	91.7	91.9
	FN Rate	22	9.4	10.4	8.4	9.8	9	8.4	8.4	8
	FP Rate	22	9.4	10.4	8.6	9.8	9	8.6	8.2	8.2
	Precision	78	90.6	89.6	91.42	90.2	91	91.42	91.78	91.82
	Recall	78	90.6	89.6	91.6	90.2	91	91.6	91.6	92
	F1	78	90.6	89.6	91.51	90.2	91	91.51	91.69	91.91
Few-Shot	Accuracy	82.2	91.5	90.9	93.8	91.7	91.7	92.3	92.4	93.2
	FN Rate	17.8	8.4	9.2	6.2	8.4	8.4	7.6	7.6	6.8
	FP Rate	17.8	8.6	9	6.2	8.2	8.2	7.8	7.6	6.8
	Precision	82.2	91.42	90.98	93.8	91.78	91.78	92.22	92.4	93.2
	Recall	82.2	91.6	90.8	93.8	91.6	91.6	92.4	92.4	93.2
	F1	82.2	91.51	90.89	93.8	91.69	91.69	92.31	92.4	93.2

**Table 2:** Performance of Models on SDCNL Dataset (Accuracy, FN rate, FP rate, Precision, Recall, and F1-score in Zero-Shot and Few-Shot learning).

Phase	Metric	Gemma	Llama	BERT	Roberta	DistilBERT	XLNet	T5	DeBERTa	Electra
Zero-Shot	Accuracy	72.1	81.4	73.8	75.4	78.9	73	73.3	75.2	73.8
	FN Rate	28	18.6	26.2	24.6	21.2	27	26.8	24.8	26.2
	FP Rate	27.8	18.6	26.2	24.6	21	27	26.6	24.8	26.2
	Precision	72.14	81.4	73.8	75.4	78.96	73	73.35	75.2	73.8
	Recall	72	81.4	73.8	75.4	78.8	73	73.2	75.2	73.8
	F1	72.07	81.4	73.8	75.4	78.88	73	73.27	75.2	73.8
Few-Shot	Accuracy	75.6	85.1	76.8	79.5	81.4	76.4	75.9	79	77.4
	FN Rate	24.4	14.8	23.2	20.4	18.6	23.6	24	21	22.6
	FP Rate	24.4	15	23.2	20.6	18.6	23.6	24.2	21	22.6
	Precision	75.6	85.03	76.8	79.44	81.4	76.4	75.85	79	77.4
	Recall	75.6	85.2	76.8	79.6	81.4	76.4	76	79	77.4
	F1	75.6	85.11	76.8	79.52	81.4	76.4	75.92	79	77.4

**Table 3:** Performance of Models on CSSRS-Suicide Dataset (Accuracy, FN rate, FP rate, Precision, Recall, and F1-score in Zero-Shot and Few-Shot learning).

Phase	Metric	Gemma	Llama	BERT	Roberta	DistilBERT	XLNet	T5	DeBERTa	Electra
Zero-Shot	Accuracy	57.11	72.18	60.44	62.35	65.18	59.83	56.68	62.39	62.24
	FN Rate	41.77	22.08	39.56	27.65	29.44	40.17	43.32	27.61	23.94
	FP Rate	15.58	17.82	20.47	11.1	14.9	18.3	22.45	13.9	20.36
	Precision	62.98	80.09	50.73	72.93	66.1	54.6	47.8	68.3	64.17
	Recall	58.23	77.92	60.44	72.35	70.56	59.83	56.68	72.39	76.06
	F1	60.51	79.97	55.24	72.85	68.18	57.96	51.87	70.32	69.61
Few-Shot	Accuracy	60.39	76.4	64.14	65.83	70.05	63.35	61.73	66.28	66.96
	FN Rate	32.46	18.38	31.22	20.44	20.22	36.65	38.27	23.72	18.25
	FP Rate	9.2	12.15	14.3	8.05	10.1	13.55	16.9	10.55	16.62
	Precision	69.08	87.89	58.12	77.6	73.4	61.22	54.9	74	67.29
	Recall	67.54	81.62	68.78	79.56	79.78	63.35	61.73	76.28	81.75
	F1	68.3	84.54	62.79	78.57	76.33	62.04	58.06	75.03	73.82

**Table 4:** Performance of Models on DepSeverity Dataset (Accuracy, FN rate, FP rate, Precision, Recall, and F1-score in Zero-Shot and Few-Shot learning).

Phase	Metric	Gemma	Llama	BERT	Roberta	DistilBERT	XLNet	T5	DeBERTa	Electra
Zero-Shot	Accuracy	63.8	75.9	66.1	66.8	70	62.1	60.5	68.2	67.4
	FN Rate	36.2	24	34	33.2	30	38	39.6	31.8	32.6
	FP Rate	36.2	24.2	33.8	33.2	30	37.8	39.4	31.8	32.6
	Precision	63.8	75.85	66.13	66.8	70	62.12	60.52	68.2	67.4
	Recall	63.8	76	66	66.8	70	62	60.4	68.2	67.4
	F1	63.8	75.92	66.07	66.8	70	62.06	60.46	68.2	67.4
Few-Shot	Accuracy	68	78.4	69.2	71.2	73.6	66.7	65.1	72.1	71.3
	FN Rate	32	21.6	30.8	28.8	26.4	33.2	34.8	28	28.8
	FP Rate	32	21.6	30.8	28.8	26.4	33.4	35	27.8	28.6
	Precision	68	78.4	69.2	71.2	73.6	66.67	65.07	72.14	71.34
	Recall	68	78.4	69.2	71.2	73.6	66.8	65.2	72	71.2
	F1	68	78.4	69.2	71.2	73.6	66.73	65.13	72.07	71.27

**Table 5:** Performance of Models on Dreaddit Dataset (Accuracy, FN rate, FP rate, Precision, Recall, and F1-score in Zero-Shot and Few-Shot learning).

Phase	Metric	Gemma	Llama	BERT	Roberta	DistilBERT	XLNet	T5	DeBERTa	Electra
Zero-Shot	Accuracy	51	66.4	51.9	52.8	61.4	51.3	46.4	51.9	51.4
	FN Rate	49	33.6	48	47.2	38.6	48.8	53.6	48	48.6
	FP Rate	49	33.6	48.2	47.2	38.6	48.6	53.6	48.2	48.6
	Precision	51	66.4	51.9	52.8	61.4	51.3	46.4	51.9	51.4
	Recall	51	66.4	52	52.8	61.4	51.2	46.4	52	51.4
	F1	51	66.4	51.95	52.8	61.4	51.25	46.4	51.95	51.4
Few-Shot	Accuracy	54	71.5	55.6	56.7	65.7	54.1	49.6	55	54.8
	FN Rate	46	28.4	44.4	43.2	34.4	46	50.4	45	45.2
	FP Rate	46	28.6	44.4	43.4	34.2	45.8	50.4	45	45.2
	Precision	54	71.46	55.6	56.69	65.73	54.11	49.6	55	54.8
	Recall	54	71.6	55.6	56.8	65.6	54	49.6	55	54.8
	F1	54	71.53	55.6	56.74	65.67	54.05	49.6	55	54.8

**Table 6:** Stress Analysis Examples and Personalized Recommendations Across Datasets.

Dataset Instance	Stress Classification	Recommendation
SAD:	Classified as Moderate Stress. LLAMA 3 detected signs of social anxiety and self-perceived judgment.	Suggested exposure therapy techniques, journaling thoughts, and consulting a therapist specializing in social anxiety.
“I feel so nervous and judged every time I’m in a group, even at work.”		
Dreaddit:	Classified as Severe Stress. LLAMA 3 identified escalating distress and potential sleep-related issues.	Recommended seeking immediate counseling, practicing relaxation techniques, and addressing sleep hygiene.
“I’ve been feeling overwhelmed and can’t sleep. It’s getting worse each day.”		
DepSeverity:	Classified as Severe Depression. LLAMA 3 recognized patterns of de- pressive severity and potential lethargy.	Suggested consulting a psychiatrist for medication, maintaining a routine, and engaging in physical activity.
“I feel hopeless and tired all the time, like there’s no point to anything.”		
SDCNL:	Classified as Moderate Stress. LLAMA 3 analyzed role conflict and emotional exhaustion.	Suggested creating a structured schedule, setting realistic goals, and engaging in mindfulness practices.
“I’m constantly stressed out about balancing work and family, it’s exhausting.”		
CSSRS-Suicide:	Classified as High Suicide Risk. LLAMA 3 flagged suicidal ideation and urgency for intervention.	Recommended contacting a crisis helpline, involving trusted individuals, and scheduling a mental health evaluation.
“I sometimes think people would be better off without me.”		

## Data Availability

The datasets used in this study are publicly available and can be accessed through the following sources: SAD [54], Dreaddit [55], DepSeverity [56], SDCNL [57], and CSSRS-Suicide [58]. No new data was generated or collected for this study.
